# Impact of Microstructure on the In Situ Formation of LDH Coatings on AZ91 Magnesium Alloy

**DOI:** 10.3390/ma18051178

**Published:** 2025-03-06

**Authors:** Nan Wang, Yulai Song, Anda Yu, Yong Tian, Hao Chen

**Affiliations:** Key Laboratory of Automobile Materials, Ministry of Education, College of Materials Science and Engineering, Jilin University, Changchun 130022, China; wnan22@mails.jlu.edu.cn (N.W.);

**Keywords:** AZ91 magnesium alloy, layered double hydroxides (LDHs), β-Mg_17_Al_12_ phase, corrosion resistance, heat treatment

## Abstract

Layered Double Hydroxide (LDH) coatings were synthesized on as-cast, T4 (solution treatment), and T6 (aging treatment) AZ91 magnesium alloys using a hydrothermal method. XRD (X-Ray Diffraction) and SEM (Scanning Electron Microscope) analyses showed that the large β-phases in as-cast AZ91 initially promoted LDH growth via galvanic corrosion, but later compromised coating integrity. In contrast, T6 and T4 alloys, with refined microstructures, formed uniform and compact LDH coatings. Corrosion resistance was enhanced in T6 and T4 alloys, as evidenced by higher impedance from EIS (Electrochemical Impedance Spectroscopy), and HER (Hydrogen Evolution Reaction) tests, due to the formation of dense LDH layers.

## 1. Introduction

Magnesium alloy, as an indispensable engineering material [1], possesses excellent physical and chemical properties [2,3], and is widely used in the transportation sector [4], 3C [5] (computer, communication, and consumer electronics), biomedical fields [6,7], and the energy industry. Due to the high electrochemical activity of magnesium [8], it is highly susceptible to corrosion in humid environments, limiting the broad application of magnesium alloys in various fields. Therefore, to enhance the corrosion resistance of magnesium alloys, surface treatment methods are commonly employed. Corrosion protection techniques often include anodizing [9], electroplating [10], and chemical conversion coatings [7]. Chemical conversion coatings, widely used in corrosion protection, have demonstrated significant potential, particularly with the application of layered double hydroxides (LDHs) [11,12]. Compared to traditional chemical conversion coatings, LDHs offer superior corrosion resistance. As a type of hydrotalcite-like compound, LDHs feature a unique interlayer structure with the general formula [M^I^/M^II^_(1–x)_ M^III^_(x)_(OH)_2_]x^+^[A^n–^]x/n·yH_2_O, where M^I^, M^II^, and M^III^ represent monovalent, divalent, and trivalent metal ions, respectively; A^n−^ refers to the interlayer anions, and x is the stoichiometric coefficient, typically ranging from 0.2 to 0.33 [13]. In recent years, researchers have conducted extensive studies on the application of LDHs in the field of corrosion protection for magnesium alloys. By adjusting solution compositions and preparation methods through co-precipitation, hydrothermal synthesis, reconstruction, and ion exchange techniques [14], they have been able to produce LDH coatings [15,16] with varying structures and properties. These coatings serve not only as physical barriers against corrosion but also, thanks to their ion-exchange capability [17], are capable of absorbing corrosive anions such as chloride ions (Cl^−^) from the solution and releasing interlayer anions. This dual functionality makes LDH coatings highly promising for improving the corrosion resistance of magnesium alloys [18,19,20].

It is well known that the structure and properties of chemical conversion coatings are closely related to the surface condition of the substrate. In the case of magnesium alloy AZ91, coating defects and failure are largely attributed to uneven coating growth, which is fundamentally caused by the non-uniform microstructure of the magnesium substrate. [21] In the study by X. Zhang et al. [22], it is suggested that the microstructure of magnesium alloys plays a crucial role in the formation of chemical conversion coatings. After the AZ91 magnesium alloy undergoes hot extrusion, the β-Mg_17_Al_12_ phase is refined and rearranged, which promotes the micro-galvanic effect during the formation process. This directly influences the surface condition of the alloy, thereby affecting the formation of LDH conversion coatings. Zeng et al. [23], in their study on LDH formation on the AZ31 surface, applied acid pretreatment to expose Al-Mn intermetallic particles. This approach revealed that the Al-Mn phase provided additional nucleation sites, thereby enhancing the growth of LDH coatings. N. Van Phuong et al. [24] found that β-Mg_17_Al_12_ acts as a cathodic center during the phosphating reaction, and a decrease in its volume fraction results in a reduction in coating weight during the process. Consequently, the researchers concluded that the formation and growth of zinc phosphate conversion coatings are closely linked to the phase composition of AZ91, particularly the presence of β-Mg_17_Al_12_. C. Zhang et al. [25] found that during the sandblasting pretreatment of AZ91, micro-galvanic corrosion occurs between the β-Mg_17_Al_12_ and α-Mg phases, which leads to an increased dissolution rate of Mg and accelerated H_2_ evolution. This process results in the formation of an uneven porous layer on the sample surface, thereby increasing the roughness of the intermediate layer. Yang et al. [26] controlled the morphology and surface uniformity of the β-Mg_17_Al_12_ phase by alkaline adjustment and acid activation, concluding that the pretreatment of AZ91 effectively optimized the surface coating. Previous studies have shown that the presence of secondary phases in magnesium alloy substrates can lead to uneven coatings growth, and that the morphology of surface coatings is a critical parameter affecting the performance of magnesium alloy coatings. Although previous studies by F. Z. Akbarzadeh et al. [27] have demonstrated that heat treatment of AZ91 can alter the ratio of cathodic (β-Mg_17_Al_12_) to anodic (α-Mg) areas in the substrate, thereby controlling the intensity of galvanic reactions on the substrate surface, the fine and coarse β phases have been found to lead to uneven formation of the Mg(OH)_2_ intermediate layer. This, in turn, can affect the uniformity of the hydroxyapatite coating during the electrodeposition process. The formation of chemical conversion coatings on AZ91 magnesium alloy surfaces is significantly influenced by the β-Mg_17_Al_12_ morphology and microstructure. Despite this, our understanding of how β-Mg_17_Al_12_ affects the formation process and performance optimization of LDH coatings is still limited. To address this gap, we conducted a study in which AZ91 magnesium alloy was subjected to heat treatment. This process included solution treatment (T4) and aging (T6), which helped us control the quantity and distribution of the β phase within the alloy. In this study, the quantity and distribution of the β phase in magnesium alloy AZ91 were regulated via heat treatment. Subsequently, the hydrolysis diagrams of Mg^2+^ and Al^3+^ under different pH conditions were constructed to predict the reaction conditions of layered double hydroxides (LDHs) on magnesium alloy AZ91. Following this, three different morphologies of heat-treated magnesium alloy AZ91 were subjected to hydrothermal reactions in two distinct solutions: one containing only Al^3+^ and the other containing both Mg^2+^ and Al^3+^. The growth differences of LDHs on the α-Mg and β-Mg_17_Al_12_ phases were observed to investigate the influence of the microstructure (α and β phases) of magnesium alloy AZ91 on the growth of LDH films.

## 2. Materials and Methods

### 2.1. The Melting of AZ91 Magnesium Alloy

In this experiment, the composition of the magnesium alloy is shown in Table 1, Al 8.57%, Fe less than 0.006%, Mn 0.28%, Si less than 0.06%, Zn 0.62%, and Cu less than 0.015%. First, the resistance furnace was heated to 400 °C, then the temperature was gradually increased to 680 °C using a stepwise heating method. The temperature was held until the AZ91 magnesium ingots in the crucible were completely melted. The temperature was slowly increased and stabilized at 710 °C. During this period, a protective gas mixture of CO_2_ and SF_6_ was introduced. The melt was held at this temperature for 20 min, during which it was stirred once to ensure uniform distribution of the alloy elements. Before casting, the upper layer of dross was removed with a slag scoop, and the temperature was slowly reduced to 700 °C. After two minutes of settling, the alloy was poured into a preheated metal mold.

### 2.2. Heat Treatment of AZ91

In this experimental investigation, the as-cast AZ91D magnesium alloy ingot was subjected to a dual heat treatment regimen, comprising a solution treatment (T4) and a subsequent aging treatment (T6). The solution treatment involved heating the alloy to a temperature of 415 °C for a duration of 24 h to facilitate the formation of a homogeneous solid solution, resulting in the solution-treated alloy, hereinafter denoted as T4-AZ91. This was followed by an aging treatment at a temperature of 200 °C for a period of 12 h to induce precipitation hardening, yielding the aged alloy, denoted as T6-AZ91.

Utilizing electrical discharge machining (EDM), the aforementioned magnesium alloys were sectioned into cubic specimens with edge lengths of 1 cm × 1 cm and 2 cm × 2 cm, each with a uniform thickness of 4 mm. The specimens were then refined to a mirror-like finish through polishing with 3000-grit silicon carbide abrasive paper. Subsequent to polishing, the specimens underwent ultrasonic degreasing in ethanol to eliminate any residual particulate matter. Finally, the specimens were air-dried in a cold environment to procure the pristine magnesium alloy samples, which were subsequently prepared for further analysis.

**Table 1:** Chemical composition of AZ31magnesium alloy (wt.%).

Material	Al	Zn	Mn	Si	Fe	Ni	Cu	Mg
AZ91	8.57	0.62	0.28	0.06	0.006	0.002	0.015	Bal.

### 2.3. Preparation of Mg-Al-NO_3_-LDH Coatings

All chemical reagents used in this work were of analytical grade, sourced from Sinopharm Chemical Reagent Co., Ltd. (Shanghai, China), including Mg(NO_3_)_2_⋅6H_2_O (AR, ≥99.0%), Al(NO_3_)_3_⋅9H_2_O (AR, ≥99.0%), NaNO_3_ (AR, ≥99.0%), NaOH(AR, ≥96.0%), NaCl(AR, ≥99.8%). In addition, all solutions used in this study were prepared with deionized water.

**Figure 1:**
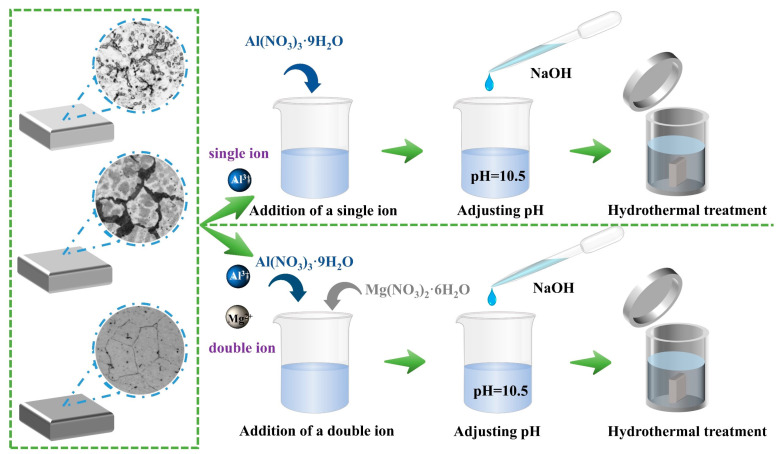
Preparation process of LDHs coating on the surface of AZ91 magnesium alloy.

In this work, a combined co-precipitation and hydrothermal growth method was employed to in situ synthesize Mg/Al-NO_3_^–^LDH coatings on the surfaces of Cast-AZ91, T4-AZ91, and T6-AZ91 magnesium alloys. Mg(NO_3_)_2_∙6H_2_O and Al(NO_3_)_3_∙9H_2_O served as the sources of Mg^2+^ and Al^3+^, respectively, in the reaction solution. The hydrothermal solutions were categorized into solution a and solution b. Solution a was prepared as a single-cation aqueous solution containing 0.01 M Al(NO_3_)_3_∙9H_2_O. Solution b was prepared as a dual-cation aqueous solution with 0.01 M Al(NO_3_)_3_∙9H_2_O and 0.02 M Mg(NO_3_)_2_∙6H_2_O for the in-situ synthesis of LDH. The Mg^2+^/Al^3+^ molar ratio was 2.0, the NO_3_^−^/Al^3+^ ratio was 2, and the OH^−^/ (Mg^2+^ + Al^3+^) ratio was 2.3; solution c was prepared by dissolving 2M NaOH in 50 mL of deionized water. Solution c was utilized to titrate solutions a and b, maintaining the pH within a range of 10.5 ± 0.5. Solution a highlights the influence of the substrate on the formation process of the LDH coating layer, while Solution b provides both Mg^2+^ and Al^3+^, which to some extent mitigate the influence of the substrate on the LDH coating layer. Once the titration was complete, the resulting reaction mixtures were transferred into a high-pressure reactor. Three different magnesium alloy specimens were placed vertically under identical conditions within the high-pressure reactor. Subsequently, the high-pressure reactor was transferred to a drying oven and the contents were subjected to heat treatment at a temperature of 393 K for specific durations: 5 min, 20 min, and 12 h, respectively, as shown in Figure 1.

Upon completion of the hydrothermal reaction, the specimens were removed from the reactor, rinsed with deionized water, and dried with cold air. The prepared specimens were labeled according to their preparation conditions. For instance, cast-AZ91 specimens treated in solution a for 5 min, 20 min, or 12 h were labeled as cast-a-5min, cast-a-20min, and cast-a-12h, respectively. The coatings in solution b were labeled as cast-b-5min, cast-b-20min, and cast-b-12h. T4-AZ91 specimens treated in solution a for 5 min, 20 min, or 12 h were designated as T4-a-5min, T4-a-20min, and T4-a-12h, respectively, while those treated in solution b were labeled as T4-b-5min, T4-b-20min, and T4-b-12h, respectively. Similarly, T6-AZ91 specimens treated in solution a for 5 min, 20 min, or 12 h were designated as T6-a-5min, T6-a-20min, and T6-a-12h, respectively, while those treated in solution b were labeled as T6-b-5min, T6-b-20min, and T6-b-12h.

### 2.4. Characterization

The distribution and structure of the secondary phases on the corroded surface of the magnesium alloy were observed using an optical microscope (Axio Lab A1 Pol, Carl Zeiss, Oberkochen, Germany). The effects of hydrothermal reaction time on the surface coatings morphology and elemental composition of AZ91, as well as the influence of the secondary phases on coatings growth, were analyzed using a field emission scanning electron microscope (FE-SEM, JEOL JSM-7900 F, JEOL Ltd. Tokyo, Japan) combined with energy dispersive X-ray spectroscopy (EDS). The crystal structure of the LDH coating layer on the AZ91 surface was determined using X-ray diffractometry (XRD, Smart Lab 9KW, Rigaku, Japan) with a Cu target (λ = 0.154 nm), employing a scanning rate of 4° min**^−^**^1^ over a 2θ range of 5° to 80°, utilizing a glancing incidence method to enhance the surface sensitivity of the analysis.

### 2.5. Electrochemical Measurement

Potentiodynamic polarization (PDP) curves and electrochemical impedance spectroscopy (EIS) of the samples were measured using an electrochemical workstation (Gamry Interface 1000E, Gamry Instruments, Warminster, PA, USA). A 10 × 10 mm surface area of the sample was exposed to a solution of 3.5% NaCl, with the remaining parts sealed in epoxy resin. Electrochemical tests were conducted in a three-electrode system using a (working electrode, GaossUnion, Wuhan, China) (test specimen), a reference electrode (saturated calomel electrode), and a counter electrode (platinum). Before testing, all samples were immersed in the test solution for at least 10 min to reach a stable state and obtain a stable open-circuit potential (OCP). Potentiodynamic polarization curves were scanned from the OCP toward both positive and negative directions at a scan rate of 1 mV/s. Furthermore, the EIS measurements were conducted from 100 kHz to 10 mHz by applying a sinusoidal amplitude perturbation of 10 mV. PDP and EIS data were analyzed using CView (Scribner Associates Inc., Southern Pines, NC, USA) and ZSimpWin (AMETEK Scientific Instruments, Oak Ridge, TN, USA), respectively. All experiments were repeated three times to ensure the reliability of the results.

### 2.6. Hydrogen Test

The hydrogen evolution experiment was conducted in 3.5 wt% NaCl at room temperature. The sample was immersed in the corrosive medium with an exposed area of 1 cm^2^, while the remaining part was sealed with epoxy resin. The hydrogen generated was collected using a homemade setup consisting of a beaker, an inverted funnel, and an inverted burette connected to the funnel. The total duration of the experiment was 240 h. To ensure the reliability of the experimental data, at least three identical samples were used under each experimental condition for comparison.

## 3. Results

### 3.1. AZ91 Phase Composition

**Figure 2:**
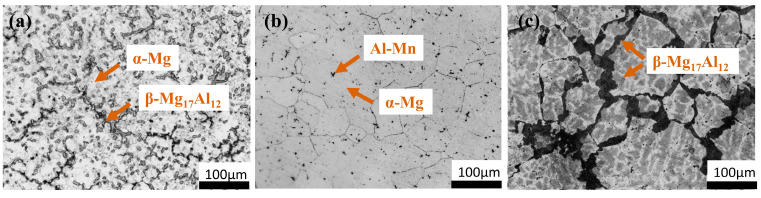
Optical microscopy of (**a**) cast-AZ91, (**b**) T4-AZ91, (**c**) T6-AZ91.

Figure 2 presents the metallographic microstructures of AZ91 magnesium alloy subjected to different heat treatments (cast-AZ91, T4-AZ91, T6-AZ91). The microstructure of cast-AZ91 consists of primary α-Mg dendrites and large-sized skeleton-like partially divorced eutectic β-Mg_17_Al_12_ phases, discontinuously distributed along the α-Mg grain boundaries. Additionally, small amounts of fine lamellar α + β eutectic and minor intermetallic AlMn phases are present. After solution treatment, the β-Mg_17_Al_12_ phase is successfully dissolved into the α-Mg matrix, resulting in the T4-AZ91 magnesium alloy comprising a single α phase and a small amount of AlMn intermetallic compounds. After aging treatment, the microstructure of T6-AZ91 magnesium alloy consists of an α-Mg matrix with short rod-shaped β-Mg_17_Al_12_ phases, both continuously and discontinuously precipitated along grain boundaries and in the grains.

### 3.2. XRD Patterns of the Coatings

**Figure 3:**
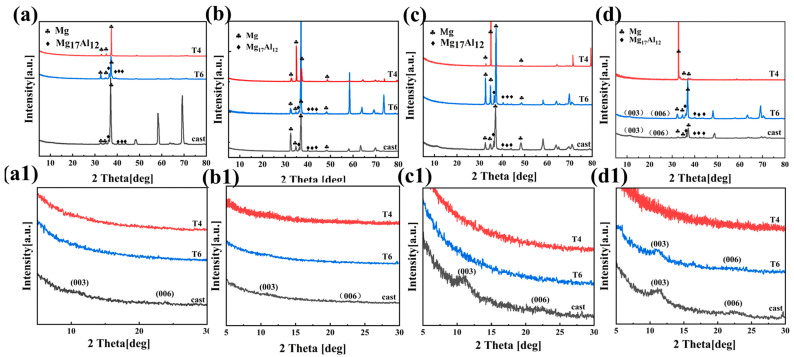
XRD patterns on (003) and (006) peaks: (**a**) cast-a-5min, T4-a-5min, T6-a-5min; (**b**) cast-a-20min, T4-a-20min, T6-a-20min; (**c**) cast-b-5min, T4-b-5min, T6-b-5min; (**d**) cast-b-20min, T4-b-20min, T6-b-20min; (**a1**–**d1**) is magnified view of (**a**–**d**) at 5–30°.

Figure 3(a–d) display the XRD patterns of the AZ91 magnesium alloy, which were obtained after the alloy was subjected to three different heat treatments following 5 min and 20 min of reaction in solutions a and b, respectively. The glancing incidence method was employed to enhance the surface sensitivity of the analysis. The X-ray diffraction analyses of the surface coatings of cast-AZ91, T4-AZ91, and T6-AZ91 in Figure 3(a–d) show that all samples exhibit diffraction peaks corresponding to the phases of the substrate alloy. This phenomenon may be attributed to the thin coatings generated in a short time, allowing X-rays to penetrate the coatings and detect the structural information of the substrate alloy. As seen from Figure 3(a,a1), after cast-AZ91 undergoes hydrothermal reaction in solution a for 5 min, the characteristic peaks of LDH appear on its surface, with 2θ angles of the (003) and (006) [28] planes at 11.1° and 21.8°, respectively. Figure 3(b,b1) show that after cast-AZ91 reacts in solution a for 20 min, the LDH characteristic peaks (003) and (006) appear, with 2θ angles of 10.9° and 21.2°, respectively. However, due to the thin LDH coating at this time, the diffraction peak intensities are relatively weak. Nevertheless, LDH was not detected in the samples T4-a-5min, T6-a-5min, T4-a-20min, and T6-a-20min. Figure 3(c,c1) indicate that after cast-AZ91 reacts in solution b for 5 min, LDH characteristic peaks (003) and (006) are detected, whereas LDH was not detected on the surfaces of T4-b-5min and T6-b-5min. Figure 3(d,d1) show that after cast-AZ91 and T6-AZ91 undergo hydrothermal reaction in solution b for 20 min, the characteristic peaks of (003) and (006) are formed on their surfaces. However, LDH was not detected in T4-a-20min. From the above results, it can be concluded that under the same preparation conditions, during the initial stage of coatings formation, the surface of cast-AZ91 is more conducive to the formation of LDH, followed by T6-AZ91, while T4-AZ91 exhibits the weakest coating-forming ability.

### 3.3. Microstructure

**Figure 4:**
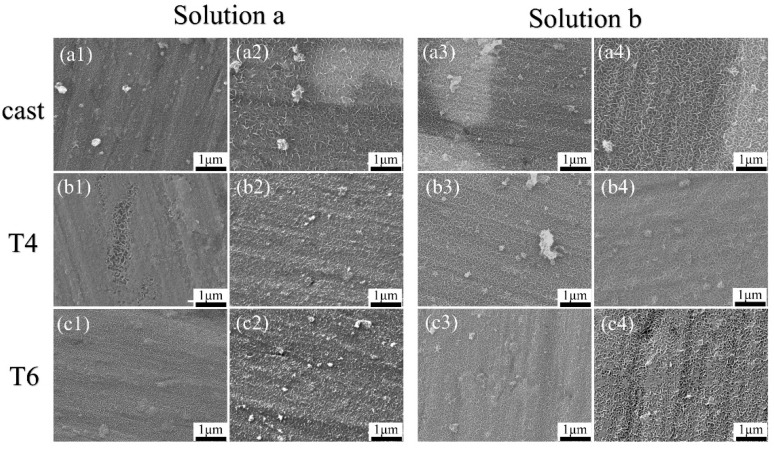
The morphology of coatings formed on magnesium (**a1**–**a4**) Cast-AZ91 samples: (**a1**) Cast-a-5 min, (**a2**) Cast-a-20 min, (**a3**) Cast-b-5 min, (**a4**) Cast-b-20 min; (**b1**–**b4**) T4-AZ91 samples: (**b1**) T4-a-5 min, (**b2**) T4-a-20 min, (**b3**) T4-b-5 min, (**b4**) T4-b-20 min; (**c1**–**c4**) T6-AZ91 samples: (**c1**) T6-a-5 min, (**c2**) T6-a-20 min, (**c3**) T6-b-5 min, (**c4**) T6-b-20 min.

Figure 4 shows the microstructures of the coatings on the AZ91 alloy after heat treatment, with reactions observed at 5 min and 20 min. As seen from Figure 4(a1–c1), after 5 min of hydrothermal reaction in solution a, all three alloys are covered by thin coatings composed of fine grains; however, visible substrate scratches indicate that the coatings are extremely thin. This suggests that during the initial stage of LDH growth, LDH coatings are formed on both the α-Mg and β-Mg_17_Al_12_. Figure 4(a2–c2) show the SEM images of cast-a-20min, T4-a-20min, and T6-a-20min. It can be observed that the surface grain size of cast-a-20min is the largest, T6-a-20min has slightly smaller grains, and T4-a-20min has the smallest grain size. Furthermore, with the extension of hydrothermal reaction time, during the process where cast-AZ91, T4-AZ91, and T6-AZ91 react in solution a from 5 min to 20 min (as illustrated in Figure 4(a1–c1) transitioning to Figure 4(a2–c2)), the grain size of the coating exhibits a significant growth trend. The coating thickness has significantly increased, and the substrate scratches have become indistinct. Figure 4(a3–c3) display the surface coatings formed on cast-AZ91, T4-AZ91, and T6-AZ91 after reacting in solution b for 5 min. LDH coatings are formed on both the α-Mg and β-Mg_17_Al_12_ phases. Compared with the samples in Figure 4(a1–c1), the substrate scratches are slightly covered. The coating of cast-b-5min (Figure 4(a3)) exhibits larger grain sizes compared to T4-b-5min (Figure 4(b3)) and T6-b-5min (Figure 4(c3)). Figure 4(a4–c4) show the morphologies of the cast-b-20min, T4-b-20min, and T6-b-20min. The grain size of cast-b-20min is the largest, followed by T6-b-20min with slightly smaller grains, while T4-b-20min has the smallest grain size.

With the extension of hydrothermal reaction time, the same conclusion is reached in solution b as in solution a: during the process where the hydrothermal reaction extends from 5 min to 20 min (as shown in Figure 4(a3–c3) transitioning to Figure 4(a4–c4)), the grain size of the coating exhibits a significant growth trend. Obviously, the coating grain size of cast-b-20min is larger. Therefore, in the initial stage of coating growth, the coating on the surface of the cast-AZ91 alloy exhibits a significant growth advantage in both solution a and solution b, and this advantage is more pronounced in the single-ion solution a. This result is consistent with the XRD analysis.

**Figure 5:**
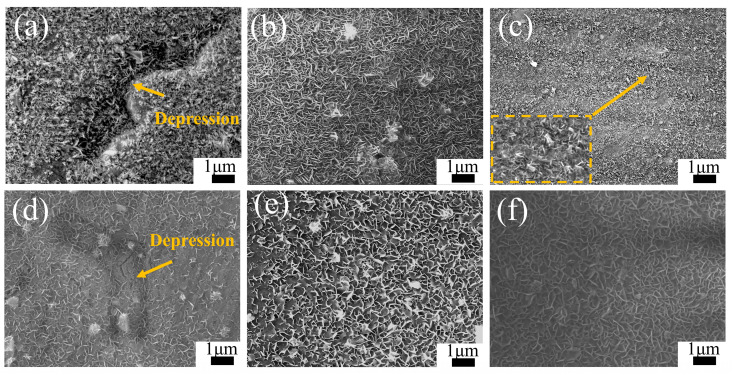
The morphology of LDH coatings formed on the magnesium: (**a**–**c**) Cast-a-12h, T4-a-12h, T6-a-12h; (**d**–**f**) Cast-b-12h, T4-b-12h, T6-b-12h. The arrows in (**a**,**d**) point to the β-phase depression areas of the coating, and the square area in (**c**) is the magnified region of the arrowed parts.

To investigate the effect of substrate microstructure on the later stages of LDH coating formation, Figure 5 presents the microstructures of coatings on the heat-treated AZ91 magnesium alloys after 12 h of hydrothermal reaction in solutions a and b. The morphology observed in the figures reveals LDH coatings on the alloy surfaces, all exhibiting the classic hexagonal platelet structure. As depicted in Figure 5a, the Cast-a-12h coating uniformly envelops the surface of the alloy, exhibiting distinct depressions that correspond to the morphology of the β-grains. These depressions suggest a comparatively thinner layer of LDH coating on the β-phase regions. Furthermore, it is observed that the LDH grains present on the β-phase are notably larger in size. In Figure 5d, the depressions on the β-grains surface become shallower in the Cast-b-12h coating, indicating reduced growth differences between the LDH coatings on the α-Mg and β-Mg_17_Al_12_ phases in solution b. Figure 5b,e show that the LDH coating on T4-AZ91 grows uniformly with smaller grains in solution a, while the addition of Mg^2+^ increases grain size. Figure 5c illustrates the morphologies of the LDH coating on the surface of T6-AZ91 under single-ion solution conditions. When T6-AZ91 is used as the substrate, the LDH coating grows more densely and uniformly. The T6-a-12h coating has a smoother surface with a more uniform grain distribution compared to cast-a-12h, and finer grains compared to T4-a-12h. In solution b, Figure 5f shows larger, more uniformly distributed LDH grains due to the ample Mg^2+^ supply. In the later stages of LDH formation, the coatings on T4-AZ91 and T6-AZ91 are more uniform and compact compared to that on cast-AZ91.

**Figure 6:**
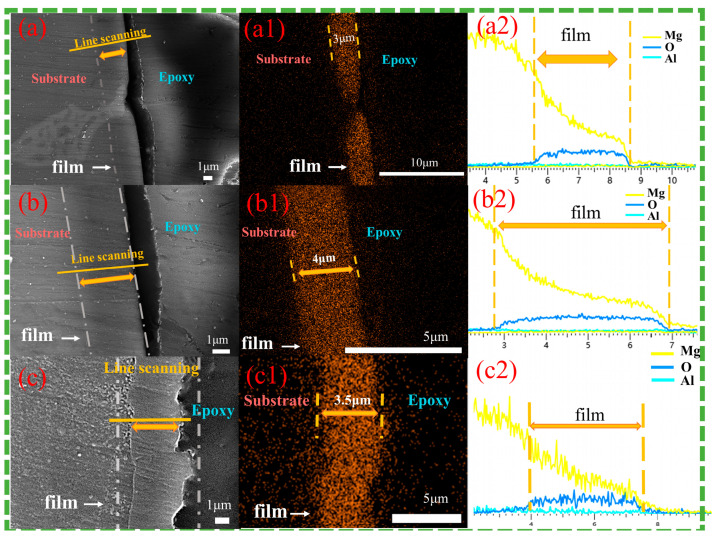
Cross-sectional morphologies (**a**–**c**) and EDS mapping results of oxygen element (**a1**–**c1**) and line scanning images (**a2**–**c2**). (**a**,**a1**,**a2**) Cast-a-12h, (**b**,**b1**,**b2**) T4-a-12h, (**c**,**c1**,**c2**) T6-a-12h.

Figure 6 shows cross-sectional images of the magnesium alloy after 12 h of hydrothermal reaction in solution a. Observations from Figure 6(a,a1) show that the Cast-a-12h appears dense, free of defects, and well-bonded to the substrate. The β phase, located in the surface and subsurface regions of the substrate, is deeply embedded in the coating, indicating that the inward growth dominates, while the outward growth remains secondary. The LDH coating thickness varies significantly between phases, with approximately 3 μm on the primary α-Mg phase, while only a very thin layer forms on the β-phase. Obviously, the coarse β-phase faces greater difficulty in supporting the growth of the LDH coating, leading to a thinner layer. As depicted in Figure 6(b,b1), the T4-a-12h coating is uniformly distributed, dense, and strongly adheres to the substrate with the thickness of 4 μm, providing excellent surface coverage and protection. Similarly, the T6-a-12h coating demonstrates uniformity and density, with an approximate thickness of 3.5 μm. The absence of a significant difference between the coatings on the β-phase and the α-Mg phase suggests that the refined and uniformly distributed β-phase encourages the formation of a complete, defect-free LDH coating, which corresponds to surface observations. Line-scan analyses from Figure 6(a2–c2) confirm the presence of Mg, Al, and O elements on both the α-Mg and β-Mg_17_Al_12_ surfaces of Cast-AZ91, as well as on the surfaces of T4-AZ91 and T6-AZ91. A gradual reduction in Mg content from the inner to outer layers of the coating indicates a decreasing Mg/Al ratio as the coating grows outward.

**Figure 7:**
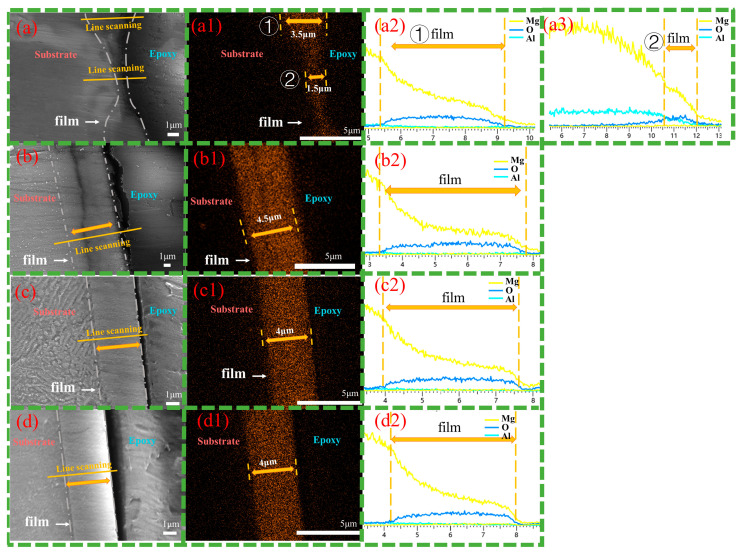
Cross-sectional morphologies (**a**–**d**) and EDS mapping results of oxygen element (**a1**–**d1**) and line scanning images (**a2**,**a3**,**b2**,**c2**,**d2**). (**a**,**a1**,**a2**,**a3**) Cast-b-12h, the marker ① represents the LDH coatings on the α-Mg matrix, while the marker ② represents the LDH coatings on the β phase. (**b**,**b1**,**b2**) T4-b-12h, (**c**,**c1**,**c2**) T6-b-12h coating grown at the grain boundary of T6-AZ91, (**d**,**d1**,**d2**) T6-b-12h coating grown at intracrystalline of T6-AZ91.

Figure 7 presents cross-sectional images of magnesium alloys after 12 h of hydrothermal reaction in solution b. Figure 7(a,a1) show that the Cast-b-12h is dense and well-bonded to the substrate. The β-phase is embedded in the coating, but at a shallower depth compared to Cast-a-12h, indicating a synergistic inward and outward growth pattern. The coating thickness varies significantly between the primary α-Mg phase and the β-phase, with the LDH layer on the α-Mg phase reaching 3.5 μm, while the β-phase coating is only 1.5 μm thick. Compared to Cast-a-12h in solution a, the coating thickness on both the α-Mg and β-Mg_17_Al_12_ has increased. Figure 7(b,b1) show the T4-b-12h coating, characterized by uniform thickness and a homogeneous, dense structure. The consistent coating of T4-b-12h, with a thickness of 4.5 μm, indicates its excellent surface coverage and protective capabilities. Compared to T4-a-12h, the same reaction time results in a thicker LDH coating. Figure 7(c,c1) and Figure 7(d,d1) show the T6-b-12h coating, with Figure 7(c,c1) displaying the LDH layer formed on the precipitated β-phase and Figure 7(d,d1) showing the LDH layer on the α-Mg surface. Compared to T6-a-12h, the coating thickness increases to around 4 μm. In T6-b-12h, there is no significant difference between the LDH coatings on the β-phase and α-Mg surfaces. The finely and uniformly distributed β-Mg_17_Al_12_ promotes the formation of a uniform, defect-free LDH layer. Additionally, the line-scan results from Figure 7(a2,a3), Figure 7(b2,c2), and Figure 7(d2) confirm the presence of Mg, Al, and O elements on both the α-phases and β-phases of Cast-AZ91, as well as on the surfaces of T4-AZ91 and T6-AZ91. The Mg content gradually decreases as the coating grows outward, eventually stabilizing. This pattern is similar to the elemental distribution observed in the coatings formed in solution a.

### 3.4. Corrosion Resistance of LDH Composite Coating

**Figure 8:**
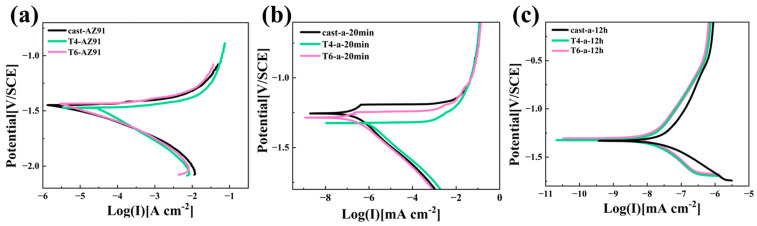
Potentiodynamic polarization curves of substrate and Mg-Al LDHs coatings: (**a**) substrate, (**b**) Cast-a-20min, T4-a-20min, T6-a-20min, (**c**) Cast-a-12h, T4-a-12h, T6-a-12h.

Figure 8 clearly presents the potentiodynamic polarization curves of three different magnesium alloy substrates and their LDH coatings after 20 min and 12 h of hydrothermal reaction. Considering the negative difference effect observed on magnesium alloy surfaces [29], in this study, only the cathodic branch of the polarization curve was analyzed, and the Tafel extrapolation method was applied to determine the relevant electrochemical parameters. The fitted data are summarized in Table 1. E_corr_ represents the corrosion potential, I_corr_ denotes the corrosion current density, and b_a_ and b_c_ refer to the anodic and cathodic Tafel slopes, respectively. Polarization resistance (R_p_) is a critical kinetic parameter in electrochemical corrosion, serving as a key indicator of corrosion rate. A higher R_p_ corresponds to a lower corrosion rate. The relationship between polarization resistance and corrosion current density is inversely proportional, which can be calculated using the well-known Stern—Geary equation [30], as shown in Equation (1)

(1) $$R_{p}=\frac{b_{a}b_{c}}{2.303i_{\mathrm{corr}}{(b}_{a}+b_{c})}$$

Figure 8a and Table 2 demonstrate that the corrosion resistance of the three magnesium alloy substrates follows the order Cast-AZ91 > T6-AZ91 > T4-AZ91. T4-AZ91 exhibits a relatively high corrosion potential and a lower corrosion current density, with an *I_corr_* value of 2.82 × 10^−5^ A·cm^−2^, which is about one order of magnitude higher than that of Cast-AZ91 and T6-AZ91. The polarization resistance (*R_p_*) of T4-AZ91 is 3.77 × 10^2^ Ω·cm^2^, which is significantly lower than that of Cast-AZ91 and T6-AZ91, indicating its poorer corrosion resistance. As shown in Figure 8b and Table 1, Cast-a-20min, T4-a-20min, and T6-a-20min exhibit a significant increase in corrosion potential and a notable reduction in I_corr_ compared to the substrate. The I_corr_ of T6-a-20min is comparable to that of Cast-a-20min but approximately two orders of magnitude lower than T4-a-20min. In terms of polarization resistance (R_p_), Cast-a-20min reaches 1.80 × 10^5^ Ω·cm^−2^, while T6-a-20min achieves 1.70 × 10^5^ Ω·cm^−2^. Both coatings demonstrate good corrosion resistance after 20 min of hydrothermal treatment. According to the analysis from Figure 4, this can be attributed to the rapid coating formation observed for Cast-a-20min and T6-a-20min. With extended hydrothermal reaction time, Figure 8c and Table 1 reveal that T4-a-12h and T6-a-12h exhibit comparable R_p_ values of 8.86 × 10^6^ Ω·cm^−2^ and 7.95 × 10^6^ Ω·cm^−2^, alongside I_corr_ values of 2.61 × 10^−9^ A·cm^−2^ and 3.41 × 10^−9^ A·cm^−2^, respectively. Conversely, the R_p_ of Cast-a-12h is 2.72 × 10^6^ Ω·cm^−2^, and the I_corr_ is 1.52 × 10^−8^ A·cm^−2^, demonstrating the poorest corrosion resistance after 12 h of hydrothermal treatment. Analysis of the cross-sectional morphologies in Figure 6a–c suggests that this is likely due to variations in coating integrity.

**Figure 9:**
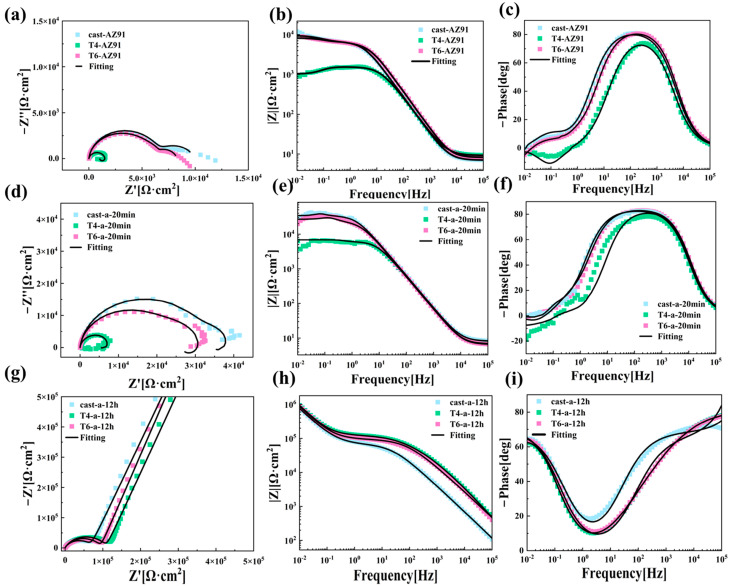
EIS results: (**a**–**c**) Cast-AZ91, T4-AZ91, T6-AZ91; (**d**–**f**) Cast-a-20min, T4-a-20min, T6-a-20min; (**g**–**i**) Cast-a-12h, T4-a-12h, T6-a-12h.

To further investigate the corrosion behavior of LDH coatings on the surfaces of magnesium alloys under three different heat treatment conditions, Figure 9 presents the EIS test results for Cast-AZ91, T4-AZ91, and T6-AZ91 after immersion in a 3.5% NaCl solution. Figure 9(a), Figure 9(d) and Figure 9(g) depict the Nyquist plots of the LDH coatings on the alloy surfaces. In Figure 9a, Cast-AZ91, T4-AZ91, and T6-AZ91 exhibit one high-frequency capacitive arc, one mid-frequency capacitive arc, and one low-frequency inductive arc. Under the 20-min hydrothermal reaction conditions, as shown in Figure 9d, the capacitive arc radius of Cast-a-20min, T4-a-20min, and T6-a-20min increases relative to their respective substrates. After 12 h of hydrothermal reaction, Figure 9g reveals that the coatings on Cast-a-12h, T4-a-12h, and T6-a-12h consist of two capacitive arcs.

The low-frequency impedance modulus (|Z| at 0.01 Hz) is often used to assess the protective performance of coatings. A higher |Z| value at 0.01 Hz generally indicates better corrosion resistance of the coating, as it effectively prevents the penetration of corrosive media [31]. Figure 9(b), Figure 9(e) and Figure 9(h) display the low-frequency impedance spectra of the three metal substrates and their synthesized LDH coatings. In Figure 9b, when the hydrothermal reaction time is 20 min, the coating on T4-a-20min shows a relatively lower impedance value, whereas the LDH coatings grown on Cast-a-20min and T6-a-20min at this initial stage exhibit higher and similar impedance modulus values, suggesting better corrosion resistance for Cast-a-20min and T6-a-20min. As the hydrothermal reaction time increases, the low-frequency impedance modulus of the Cast-a-12h coating is lower compared to T4-a-12h and T6-a-12h, indicating relatively inferior corrosion resistance.

Figure 9(c), Figure 9(f) and Figure 9(i) display the Bode phase angle–frequency plots of the LDH coatings on the magnesium alloy surfaces. As seen in Figure 9f, the coatings on Cast-a-20min, T4-a-20min, and T6-a-20min exhibit three time constants. The high-frequency relaxation process corresponds to the charge and discharge of the coating, the mid-frequency relaxation process is associated with the double-layer capacitance at the solution–substrate interface, and the low-frequency relaxation process relates to pitting corrosion on the substrate surface. From the Bode phase plot in Figure 9i, it is evident that the in-situ grown LDH coatings on the magnesium alloy surface after 12 h of hydrothermal treatment exhibit two time constants.

**Figure 10:**
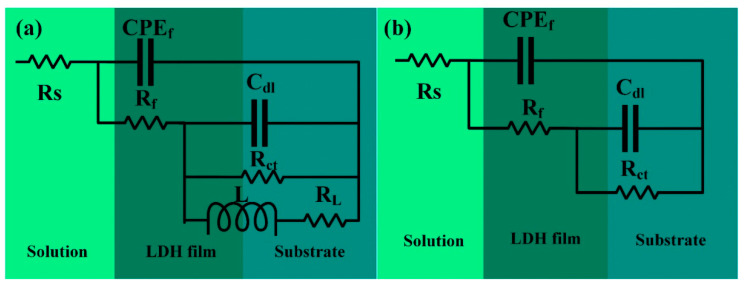
Equivalent circuits used to fit the EIS data of (**a**) Cast-AZ91, T4-AZ91, T6-AZ91 and Cast-a-20min, T4-a-20min, T6-a-20min; (**b**) Cast-a-12h, T4-a-12h, T6-a-12h.

The EIS spectra in Figure 9 were fitted using equivalent circuits, as shown in Figure 10. The equivalent circuit in Figure 10a was used for fitting and analyzing the surface coatings on Cast-AZ91, T4-AZ91, and T6-AZ91 after 20 min of hydrothermal treatment. Figure 10b was applied to the fitting analysis of Cast-a-12h, T4-a-12h, and T6-a-12h. The corresponding fitting results are presented in Table 2, including coating capacitance (CPE_f_), coating resistance (R_f_), double-layer capacitance (CPE_dl_), charge transfer resistance (R_ct_), inductance (L), and inductive resistance (R_L_). To account for characteristics such as surface roughness and porosity, a constant phase element (CPE) was used instead of a pure capacitor during fitting [32], to compensate for the system’s non-uniformity. The impedance of the constant phase element is expressed by Equation (2), where Y_0_ is always positive and n (0 < n < 1) is the dispersion index. The closer the value of n is to 1, the more the behavior of the capacitance approaches that of an ideal pure capacitor. The capacitance (C) can be calculated using Equation (3) [33].

(2) $$Z_{\mathrm{CPE}}=\frac{1}{Y_{0}{\left(j\omega \right)}^{n}}$$

(3) $$C={Y_{0}}^{\frac{1}{n}}R^{\frac{1-n}{n}}$$

As shown in the results of Table 3, R_ct_ indicates the ease of charge transfer at the substrate and solution interface during electrochemical reactions. R_f_ is used as a criterion for evaluating the compactness of the coating [34], while C_f_ represents the extent of electrolyte penetration into the coating [35]. Based on the Rct values of the three magnesium alloys, T4-AZ91 exhibits inferior corrosion resistance, with an R_ct_ value of 1547 Ω·cm^2^. After 20 min of hydrothermal treatment, T4-a-20min shows the lowest corrosion resistance, with an R_f_ of 6172 Ω·cm^2^, an R_ct_ of 1680 Ω·cm^2^, and a C_f_ of 3.20 × 10^−6^ F·cm^−2^. This indicates that the T4-a-20min coating is thinner, less compact, and more prone to pitting corrosion due to electrolyte penetration, whereas Cast-a-20min exhibits the best corrosion resistance. At a hydrothermal reaction time of 12 h, the R_f_ and R_ct_ values of Cast-a-12h, T4-a-12h, and T6-a-12h show significant improvement, indicating that with prolonged hydrothermal treatment, both the thickness and compactness of the LDH coatings on the surfaces of the three magnesium alloys increase. However, the R_f_ (7.06 × 10⁴ Ω·cm^2^) and R_ct_ (8.48 × 10^8^ Ω·cm^2^) values for Cast-a-12h are comparatively lower, suggesting that the uniformity and compactness of the Cast-a-12h coating are inferior. Additionally, the relatively large C_f_ value (9.8 × 10^−8^ F·cm^−2^) further indicates that the Cast-a-12h coating is more prone to electrolyte penetration during immersion. According to Figure 3(b,b1) and Figure 4(a2–c2), in the early stages of LDH coating growth, a relatively thin LDH layer initially forms on the Cast-AZ91 surface, displaying superior corrosion resistance in the electrochemical impedance spectroscopy (EIS) tests. As the hydrothermal reaction time increases, T4-a-12h and T6-a-12h exhibit better corrosion resistance compared to Cast-a-12h. As shown in Figure 5a and Figure 6a, during the later stages of LDH coating growth, the coarse β-phase in Cast-AZ91 disrupted the integrity of the LDH coating, resulting in uneven growth and lower compactness. In contrast, T4-a-12h and T6-a-12h exhibited more uniform growth and better corrosion resistance in the final stages.

### 3.5. Hydrogen Evolution Tests

**Figure 11:**
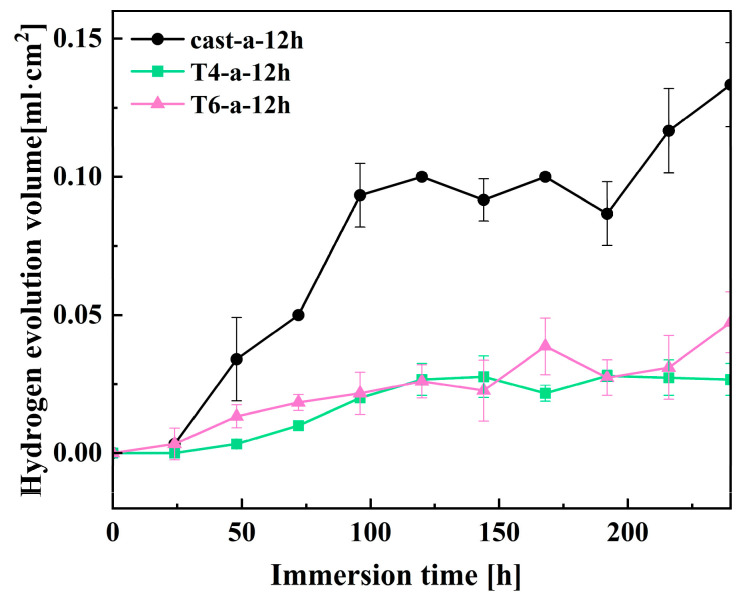
Hydrogen evolution volume of 12h in-situ LDH on magnesium.

The hydrogen evolution experiment was conducted to further analyze the effect of hydrothermal reaction time on the corrosion performance of magnesium alloys under three different heat treatment conditions in solution a. Figure 11 shows the hydrogen evolution volume over time for different samples immersed in 3.5 wt% NaCl solution for 240 h. During magnesium corrosion, hydrogen gas evolves as a byproduct, and the electrochemical process can be summarized by the following equation [36].

Anodic reaction:

(4) $$\mathrm{Mg}\to {\mathrm{Mg}}^{2+}+2e^{-}$$

Cathodic reaction:

(5) $$2H_{2}O+2e^{-}⟶H_{2}+{2\mathrm{OH}}^{-}$$

Overall reaction:

(6) $$\mathrm{Mg}+2H_{2}O⟶{\mathrm{Mg}\left(\mathrm{OH}\right)}_{2}+H_{2}↑$$

From the overall reaction equation, it can be observed that the dissolution of 1 mol of magnesium corresponds to the release of 1 mol of hydrogen gas. Therefore, the volume of hydrogen released can indirectly reflect the corrosion resistance of the magnesium alloy surface coating. Prolonged immersion in the solution results in damage to the LDH coating on the surface of the magnesium alloy, with the Cast-a-12h coating exhibiting a significantly larger volume of hydrogen gas release. This is likely due to the uneven coating of the Cast-a-12h sample, allowing the corrosive medium to penetrate the substrate through small pores and defects. After 240 h of immersion, the hydrogen evolution volume for Cast-a-12h reached 0.13 mL·cm^−2^. Notably, the hydrogen evolution volumes for the T6-a-12h and T4-a-12h coatings were lower compared to Cast-a-12h, indicating better corrosion resistance. This result is consistent with previous findings from electrochemical impedance spectroscopy (EIS) and polarization curve tests.

## 4. Discussion

### 4.1. Free Energy Estimation and Formation Mechanism of LDH Coating

**Figure 12:**
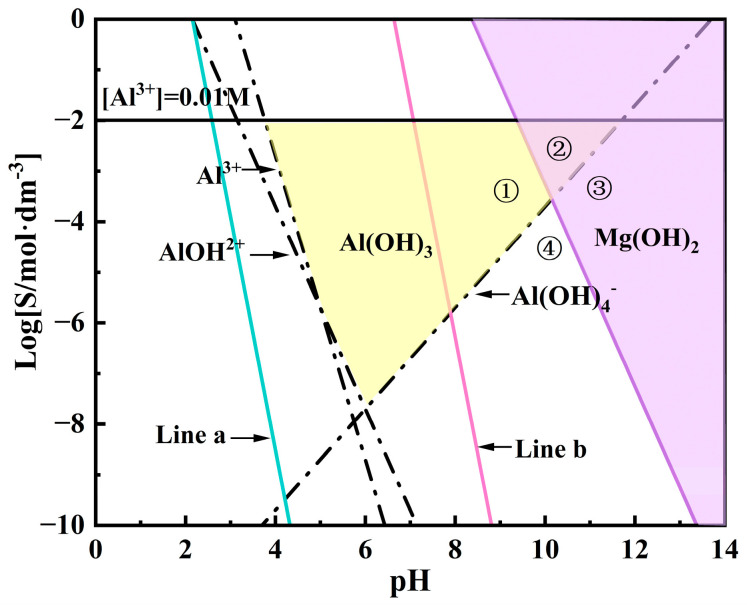
Domain diagram of Al(OH)_3_, Mg(OH)_2_, and Mg/Al-NO_3_-LDH (The yellow area represents Al(OH)_3_, while the purple area represents Mg(OH)_2_. Line a represents the LDH solubility line for x = 0.33, while Line b represents the LDH solubility line for x = 0.2; Region ① represents the reaction area of Al(OH)_3_ with Mg^2+^, Region ② represents the reaction area of Al(OH)_3_ with Mg(OH)_2_, Region ③ represents the reaction area of Mg(OH)_2_ with Al(OH)_4_^−^, and Region ④ represents the reaction area of Mg^2+^ with Al(OH)_4_^−^).

In the application of LDH, its stability is typically assessed by its solubility in aqueous solutions, but the variation in interlayer ions makes its solubility difficult to predict. Therefore, Allada et al. [37] used the enthalpy of formation of single cation components in LDH as a stability indicator for different anion insertions. This serves as an indicator of the stability of hydrotalcite-like mechanical mixtures, linking LDH stability with water solubility. By calculating the mechanical mixtures of binary compounds formed by the elements in LDH (i.e., hydroxides and carbonates), the enthalpy and free energy of formation of LDH can be estimated. The formation process is represented by Equations (7) and (8), respectively [38]. In this study, the Gibbs free energy of the synthesized LDH was estimated based on the conclusions above, as the sum of the Gibbs free energies of Mg(OH)_2_, Al(OH)_3_, and Mg(NO_3_)_2_. The reaction free energy of Mg/Al-NO_3_-LDH can be calculated from Equations (7) and (8). According to current research, the parameter x for LDH is selected within the range of 0.2 to 0.33 [39]. The estimated results show that when x = 0.2, at 393K, ΔG_Mg/Al-NO3-LDH_ is approximately −113.81 KJ/mol, and when x = 0.33, ΔG_Mg/Al-NO3-LDH_ is approximately −143.84 KJ/mol at the same temperature. Furthermore, the equilibrium constants for Mg^2+^ and Al^3+^ ions in the system can be calculated using Equations (10) and (11) [40]. In this study, the trivalent cation parameter x in LDH was set to 0.2 and 0.33. Based on Equations (10) and (11), the sum of the logarithmic values of the metal ion solubilities was plotted against pH to form a straight line. In Figure 12, Line a represents the variation of S_Mg/Al-NO3-LDH_ with pH when x = 0.2, while Line b corresponds to x = 0.33.

(7) $$\left(1-\frac{3x}{2}\right)\mathrm{Mg}{\left(\mathrm{OH}\right)}_{2}\left(s\right)+\frac{x}{2}\mathrm{Mg}{\left({\mathrm{NO}}_{3}\right)}_{2}\left(s\right)+x{\mathrm{Al}\left(\mathrm{OH}\right)}_{3}\left(s\right)+mH_{2}O\left(l\right)\to {\mathrm{Mg}}_{1-x}{\mathrm{Al}}_{x}{\left(\mathrm{OH}\right)}_{2}{{(\mathrm{NO}}_{3})}_{x}\cdot mH_{2}O$$

(8) $${\mathrm{Mg}}_{1-x}{\mathrm{Al}}_{x}{\left(\mathrm{OH}\right)}_{2}{{(\mathrm{NO}}_{3})}_{x}\cdot mH_{2}O+2H^{+}\to \left(1-x\right){\mathrm{Mg}}^{2+}+x{\mathrm{Al}}^{3+}+x{{\mathrm{NO}}_{3}}^{-}+\left(2+m\right)H_{2}O$$

(9) $$∆G_{r}=-\mathrm{RTLnK}$$

(10) $$\mathrm{Log}K_{0.2}=0.8\mathrm{Log}\left[{\mathrm{Mg}}^{2+}\right]+0.2\mathrm{Log}\left[{\mathrm{Al}}^{3+}\right]+0.2\mathrm{Log}\left[{{\mathrm{NO}}_{3}}^{-}\right]+2\mathrm{Log}\left[{\mathrm{OH}}^{-}\right]$$

(11) $$\mathrm{Log}K_{0.33}=0.67\mathrm{Log}\left[{\mathrm{Mg}}^{2+}\right]+0.33\mathrm{Log}\left[{\mathrm{Al}}^{3+}\right]+0.33\mathrm{Log}\left[{{\mathrm{NO}}_{3}}^{-}\right]+2\mathrm{Log}\left[{\mathrm{OH}}^{-}\right]$$

The concentration of cations in the LDH structure at different pH values is one of the key factors influencing the LDH growth process. As shown in Figure 12, aluminum ions are amphoteric metal cations that form different hydroxides with OH^−^ under various pH conditions. The reactions and parameters for Al hydroxides used in this study are provided by the third edition of Aquatic Chemistry [41]. The specific parameters for the hydrolysis of the two cations were modified based on H. Tamura et al. [42]. In this context, K_Al_ represents the solubility product constant of aluminum hydroxide, β_1_-β_4_ are the stability constants of aluminum hydroxide complexes, K_Mg_ is the solubility product constant of magnesium hydroxide, and S_Mg_ is the solubility constant of Mg. All of these parameters are concentration constants. Using the ion product constant of water, K_w_ (10**^−^**^14^ mol^2^ dm^−6^), hydrolysis diagrams for different hydroxyl compounds formed by Mg and Al were plotted (Figure 12). The hydrolysis reaction equation is provided in Equations (12)–(25).

(12) $${\mathrm{Al}\left(\mathrm{OH}\right)}_{3}⇌{\mathrm{Al}}^{3+}+{\mathrm{OH}}^{-}$$

(13) $$K_{\mathrm{Al}}=\left[{\mathrm{Al}}^{3+}\right]{\left[{\mathrm{OH}}^{-}\right]}^{3}=10^{-32.7}{\mathrm{mol}}^{4}{\mathrm{dm}}^{-12}$$

(14) $${\mathrm{Al}}^{3+}+{\mathrm{OH}}^{-}⇌{\mathrm{AlOH}}^{2+}$$

(15) $$\beta_{1}=\frac{\left[{\mathrm{AlOH}}^{2+}\right]}{\left[{\mathrm{Al}}^{3+}\right]\left[{\mathrm{OH}}^{-}\right]}=10^{9.0}{\mathrm{mol}}^{-1}{\mathrm{dm}}^{3}$$

(16) $${\mathrm{Al}}^{3+}+2{\mathrm{OH}}^{-}⇌{\mathrm{Al}{\left(\mathrm{OH}\right)}_{2}}^{+}$$

(17) $$\beta_{2}=\frac{\left[{\mathrm{Al}{\left(\mathrm{OH}\right)}_{2}}^{+}\right]}{\left[{\mathrm{Al}}^{3+}\right]{\left[{\mathrm{OH}}^{-}\right]}^{2}}=10^{18.7}{\mathrm{mol}}^{-2}{\mathrm{dm}}^{6}$$

(18) $${\mathrm{Al}}^{3+}+3{\mathrm{OH}}^{-}⇌{\mathrm{Al}\left(\mathrm{OH}\right)}_{3}$$

(19) $$\beta_{3}=\frac{\left[A{l\left(\mathrm{OH}\right)}_{3}\left(\mathrm{aq}\right)\right]}{\left[{\mathrm{Al}}^{3+}\right]{\left[{\mathrm{OH}}^{-}\right]}^{3}}=10^{27.0}{\mathrm{mol}}^{-3}{\mathrm{dm}}^{9}$$

(20) $${\mathrm{Al}}^{3+}+4{\mathrm{OH}}^{-}⇌{{\mathrm{Al}\left(\mathrm{OH}\right)}_{4}}^{-}$$

(21) $$\beta_{4}=\frac{\left[{A{l\left(\mathrm{OH}\right)}_{4}}^{-}\right]}{\left[{\mathrm{Al}}^{3+}\right]{\left[{\mathrm{OH}}^{-}\right]}^{4}}=10^{33.0}{\mathrm{mol}}^{-4}{\mathrm{dm}}^{12}$$

(22) $$S_{\mathrm{Al}}=\frac{K_{\mathrm{Al}}\left(1+\beta_{1}\left[{\mathrm{OH}}^{-}\right]+\beta_{2}{\left[{\mathrm{OH}}^{-}\right]}^{2}+\beta_{3}{\left[{\mathrm{OH}}^{-}\right]}^{3}+\beta_{4}{\left[{\mathrm{OH}}^{-}\right]}^{4}\right)}{{\left[{\mathrm{OH}}^{-}\right]}^{3}}$$

(23) $${\mathrm{Mg}\left(\mathrm{OH}\right)}_{2}⇌{\mathrm{Mg}}^{2+}+2{\mathrm{OH}}^{-}$$

(24) $$K_{\mathrm{Mg}}={[\mathrm{Mg}}^{2+}]{\left[{\mathrm{OH}}^{-}\right]}^{2}=10^{-11.6}{\mathrm{mol}}^{3}{\mathrm{dm}}^{-9}$$

(25) $$S_{\mathrm{Mg}}=\frac{K_{\mathrm{Mg}}}{{\left[{\mathrm{OH}}^{-}\right]}^{2}}$$

In this study, we used two hydrothermal reaction solutions. The Al^3+^ concentration in solution a was 0.01M, while in solution b, the Al^3+^ concentration was 0.01M and the Mg^2+^ concentration was 0.02M. Specifically, under the conditions of solution b, the LDH parameter x was set to 0.33, and a solubility line marking the onset of LDH formation, as represented by Line b in Figure 12, was observed. Similarly, when the LDH parameter x was set to 0.2, the solubility line of LDH, represented by Line a in Figure 12, was also observed. At a pH of 10.5 and using Equations (9) and (10), we predict that, considering only thermodynamic factors, LDH can form on the magnesium alloy in both solution a and solution b. Although the synthesis of LDH generally requires a pH greater than 7 and is more stable in alkaline conditions, there are currently no studies that can precisely explain the formation mechanism of LDH during hydrothermal reactions. It is widely believed that the formation of LDH may involve divalent and trivalent metal hydroxides, and that within a specific pH range, these metal ions may undergo exchange and substitution reactions. For example, Yang et al. [43] proposed that, during the initial stage of LDH synthesis, an amorphous colloidal aluminum hydroxide first forms from the magnesium–aluminum precursor salt solution, which subsequently transforms into γ-AlOOH, with Mg^2+^ gradually incorporating into the thin layers of γ-AlOOH, playing a crucial role in LDH crystal formation. Chen et al. [44] dissolved Mg(OH)_2_ and Al(OH)_3_ in solution under weakly acidic conditions, leading to ion substitution and the formation of two coexisting LDH precursors, Mg_2_Al(OH)_7_ and MgAl_2_(OH)_8_·xH_2_O. At a pH of 10.5, the dissolution of Al^3+^ and the exchange of CO_3_^2−^ and OH^−^ lead to the formation of LDH. S. Paikaray et al. [45] used NaOH to titrate solutions containing monovalent and trivalent cations, adjusting the pH from 1.7 to 12.5, where Al(OH)_3_ precipitated first, followed by Mg(OH)_2_. During this process, it was found that adding Mg^2+^ to Al(OH)_3_ readily forms LDH. Thus, we infer that the LDH formation process is related to the hydroxides of both metals. The solubility lines of metal hydroxides at different pH values in Figure 12 aid in understanding the synthesis conditions and formation process of LDH.

Xu and Lu [46] proposed that the specific formation process of LDH under varying pH levels and ion concentrations follows the reactions outlined in Equations (26)–(29) [46]. Thus, in this study, the LDH formation mechanism was preliminarily inferred by combining the existing formation mechanisms with Equations (26)–(29) [46] and Figure 12. When only Al^3+^ is present in the solution (as in solution a), as the pH increases, Al^3+^ first precipitates to form Al(OH)_3_, which then dissolves to form Al(OH)4^−^. When a magnesium alloy is placed in solution a, the Mg matrix gradually dissolves at around pH 10.5. According to reaction (28), Al(OH)_3_ can react with Mg^2+^ to form LDH [47], as seen in Region ① of Figure 12. As the OH^−^ concentration increases, Al(OH)_4_^−^ reacts with Mg^2+^ to form LDH [48] according to reaction (27), as seen in Region ④ of Figure 12. When Mg^2+^ is introduced into the reaction solution (as in solution b), the Mg^2+^ concentration increases. Under low alkalinity, Mg(OH)_2_ reacts with Al(OH)_3_ to form LDH [49], according to reaction (26), as seen in Region ② of Figure 12. Under higher alkalinity, Mg(OH)_2_ reacts with Al(OH)_4_^−^ to form LDH [46,50], according to reaction (29), as seen in Region ③ of Figure 12. The above studies demonstrate that the hydroxide compounds of Mg and Al can both form LDH with their respective ions; however, the rate of LDH synthesis is influenced by metal ion concentration and pH values. In other words, metal ion concentration and pH play a critical role in LDH formation, as they not only determine the types of precursors and primary nuclei but also significantly affect the reaction process, making them key factors in LDH synthesis. The process of LDH formation on the surface of AZ91 and the influence of the AZ91 phase composition on LDH will be discussed in subsequent sections.

(26) $$a{\mathrm{Mg}\left(\mathrm{OH}\right)}_{2}\left(s\right)+{\mathrm{Al}\left(\mathrm{OH}\right)}_{3}\left(s\right)+xH_{2}O+A^{-}⇌{\mathrm{Mg}}_{a}A{l(\mathrm{OH})}_{2+2a}A\cdot xH_{2}O\left(s\right)+{\mathrm{OH}}^{-}$$

(27) $$a{\mathrm{Mg}}^{2+}+{\mathrm{Al}(\mathrm{OH})}_{4}^{-}+(2a-2{)\mathrm{OH}}^{-}+xH_{2}O+A^{-}⇌{\mathrm{Mg}}_{a}A{l(\mathrm{OH})}_{2+2a}\mathrm{Ax}{\cdot H}_{2}O\left(s\right)$$

(28) $$a{\mathrm{Mg}}^{2+}+{\mathrm{Al}\left(\mathrm{OH}\right)}_{3}\left(s\right)+(2a-1{)\mathrm{OH}}^{-}+xH_{2}O+A^{-}⇌{\mathrm{Mg}}_{a}A{l(\mathrm{OH})}_{2+2a}A\cdot xH_{2}O\left(s\right)$$

(29) $$a{\mathrm{Mg}\left(\mathrm{OH}\right)}_{2}\left(s\right)+{\mathrm{Al}(\mathrm{OH})}_{4}^{-}+xH_{2}O+A^{-}⇌{\mathrm{Mg}}_{a}A{l(\mathrm{OH})}_{2+2a}A\cdot xH_{2}O\left(s\right)+{2\mathrm{OH}}^{-}$$

### 4.2. Formation Process and Mechanism Analysis of LDH on AZ91

The AZ91 alloy used in this study mainly consists of an α-Mg matrix and a β-Mg_17_Al_12_ phase. The uneven distribution of Al in the primary α-phase, eutectic α-phase, and β-phase leads to microstructural heterogeneity [51]. Under self-corrosion conditions, the potential difference between the constituent phases in the magnesium alloy causes the second-phase impurities in the alloy and the magnesium matrix to act as the cathodic and anodic phases, respectively, in micro-galvanic corrosion, forming galvanic couples and generating current [52,53,54]. G.L. Song et al. [55,56,57] suggested that the micro-galvanic interaction between the α-Mg and β-Mg_17_Al_12_ phases is the primary corrosion mechanism in Mg-Al alloys, where α-Mg acts as the anode and undergoes dissolution, while hydrogen evolution occurs on the cathodic surface of β-Mg_17_Al_12_, as shown in Equations (4)–(6). Under conditions of the solution a, Cast-AZ91 contains a coarse skeletal, eutectic β-phase, as shown in Figure 2a. This large, isolated β-Mg_17_Al_12_, along with the adjacent α-Mg, forms strong galvanic corrosion due to the large cathode-to-small anode ratio, which causes rapid dissolution of α-Mg at the anode, while hydrogen evolution occurs at the β-phase cathodic surface, increasing OH^−^ concentration and pH, with H_2_ evolution at the β-phase surface. Thus, in the early stages of the reaction in solution a, Mg^2+^ concentration rapidly increases on the α-Mg surface, leading the solution composition to enter Region ① in Figure 12, and forming LDH according to reaction (28). On the β-phase surface, pH values increase due to hydrogen evolution, and Mg^2+^ concentration gradually increases due to Mg^2+^ diffusion on the α-Mg surface. This causes the solution to enter Region ④ in Figure 12, leading to the formation of LDH according to reaction (27).

In this research, the distributions of the β-phase in the magnesium alloy were altered by heat treatment. After T6 treatment, the alloy exhibited finely dispersed β-phase precipitate, as shown in Figure 2c. Although a potential difference still exists between the β and α-Mg phases, the fine and uniformly distributed β-phase act as small cathodes, promoting the uniform corrosion creating numerous micro-galvanic regions on the alloy surface, promoting the uniform corrosion of the magnesium matrix [58]. This process increases the OH^−^ concentration and raises the pH values. In the early stages of the hydrothermal reaction, T6-AZ91 undergoes uniform dissolution, with Mg^2+^ rapidly increasing due to the micro-galvanic effect. The solution composition enters Region ④ of Figure 12, forming LDH according to reaction (27). After solution treatment, T4-AZ91 becomes a single α-Mg solid solution and the coarse β-phase was eliminated, as shown in Figure 2b. In the early stages of the hydrothermal reaction, the solid solution undergoes uniform dissolution during self-corrosion, producing a small amount of Mg^2+^. The solution composition enters Region ④ of Figure 12, forming LDH according to reaction (27).

Under the conditions of solution b, the Mg^2+^ concentration is sufficient, and strong galvanic corrosion occurs between α-Mg and β-Mg_17_Al_12_ in Cast-AZ91. α-Mg, acting as the anode, undergoes rapid dissolution, causing a rapid increase in Mg^2+^ concentration. The solution composition enters Region ② of Figure 12, forming LDH according to reaction (26). Meanwhile, the β-phase acts as the cathode, with an increase in surface pH values. The sufficient Mg^2+^ concentration drives the solution composition into Region ③, where LDH forms according to reaction (29). In T6-AZ91, the widespread distribution of β-phase creates multiple micro-galvanic regions within the alloy, where α-Mg dissolves as the anode and the fine β-phase generates hydrogen evolution on its surface as the cathode. In the early stages of the reaction, this corrosion behavior results in the uniform composition of the solution on the whole surface of T6-AZ91. The solution adjacent to the solid–liquid interface contains a high concentration of OH^−^ and Mg^2+^, as shown in Region ③, where LDH forms according to reaction (29). In the initial stage of the hydrothermal reaction, the compositions of the solution on the surface T4-AZ91 are similar to T6-AZ91, namely, the concentrations of Mg^2+^ and OH^−^ are high. The LDH coating forms in Region ③, according to reaction (29).

**Figure 13:**
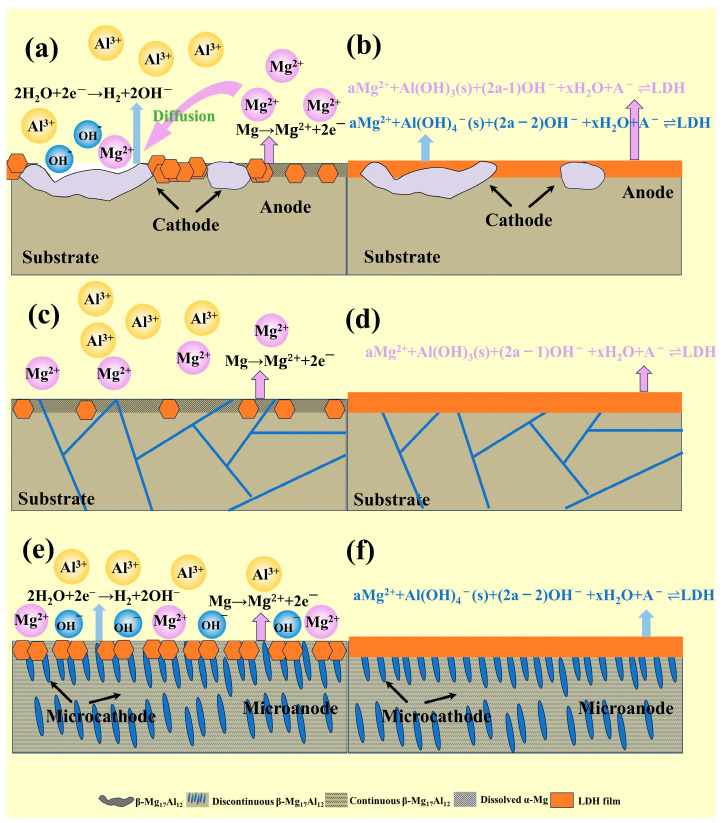
Mechanism of LDH layer formation on AZ91 matrix: (**a**) Cast-a-20min, (**b**) Cast-a-12h, (**c**) T4-a-20min, (**d**) T4-a-12h, (**e**) T6-a-20min, (**f**) T6-a-12h.

From a kinetic perspective, in solution a, the potential difference between the β-phase and α-phase in cast-AZ91 induces strong galvanic corrosion. As shown in Figure 3, anodic dissolution occurs on the α-phase surface, providing a substantial amount of Mg^2+^ for LDH coating formation. At the initial stage, LDH forms rapidly and grows extensively on the α-Mg surface. On the β-phase surface, cathodic hydrogen evolution occurs, increasing OH^−^ concentration. The Mg^2+^ required for LDH formation on the β-phase is supplied through diffusion from the dissolving α-Mg. Due to the larger size of the β-phase, the diffusion path for Mg^2+^ is longer, resulting in a lower concentration of Mg^2+^ on the β-phase surface, and consequently slower coating growth, as illustrated in Figure 13a. As the hydrothermal reaction progresses, Figure 5a and Figure 6a show that the thickness of the cast-a-12h coating varies between the α-Mg and β-Mg_17_Al_12_ phases, with the α-Mg surface having a significantly thicker coating, while the β-phase exhibits a much thinner LDH layer. This observation is consistent with the findings of X. Zhang et al. [22], as shown in Figure 13b. The coating primarily grows inward, with outward growth playing a secondary role. During LDH layer growth, the β-phase, acting as the cathode, is protected from dissolution and becomes embedded within the coating. On the other hand, the evolution of hydrogen gas from the β-phase surface inhibits coating growth, potentially leading to uneven LDH formation and increased susceptibility to damage. After T6 treatment, the finely dispersed β-phase re-precipitates in the magnesium alloy. Although micro-galvanic corrosion still occurs in T6-AZ91, the β-phase is more evenly distributed and significantly smaller compared to cast-AZ91. As shown in Figure 4(c2), during the early stages of coating formation, the β-phase forms multiple micro-galvanic corrosion sites on the magnesium alloy surface, promoting LDH layer growth. On the other hand, the finely distributed β-phase reduces the diffusion distance of dissolved Mg^2+^ in T6-AZ91, leading to uniform coating growth, as shown in Figure 13e. As the hydrothermal reaction proceeds, Figure 5c and Figure 6c indicate that the T6-a-12h coating is uniform and densely structured, as depicted in Figure 13f. As shown in Figure 4(b2), the homogeneous structure of T4-AZ91 exhibits similar behavior to T6-AZ91. During the initial stage of coating formation, T4-AZ91 undergoes uniform dissolution, gradually forming an even LDH layer on its surface, effectively covering the magnesium alloy, as shown in Figure 13c. Additionally, as the hydrothermal reaction progresses, T4-a-12h exhibits excellent surface coverage, which helps block the penetration of corrosive media, further demonstrating the integrity, uniformity, and protective properties of T4-a-12h, as shown in Figure 13d.

In solution b, the addition of Mg^2+^ creates more favorable conditions for the reaction. In the initial stage, the LDH layers shown in Figure 4(a4–c4) exhibit a noticeable growth advantage compared to those in Figure 4(a2–c2). As the reaction progresses, the substrate’s influence on the LDH layer diminishes. In Figure 5d, the surface depressions of the LDH layer in the Cast-b-12h coating are visibly reduced. Although the coarse β-phase is embedded within the layer, Figure 7a shows that its depth of embedding remains relatively shallow. Additionally, a comparison of Figure 6a and Figure 7a reveals a variation in the thickness of the LDH layers between the α-Mg and β-Mg_17_Al_12_ surfaces, with Cast-b-12h showing an increased layer thickness compared to Cast-a-12h, indicating that Cast-b-12h grows through combining the outward and inward mechanisms. Figure 5e,f and Figure 7b–d demonstrate that T6-b-12h and T4-b-12h develop uniform coatings with more substantial coverage, similar to those formed in solution a.

### 4.3. Impact of β-Mg_17_Al_12_ on Corrosion Resistance of LDH Coatings

**Figure 14:**
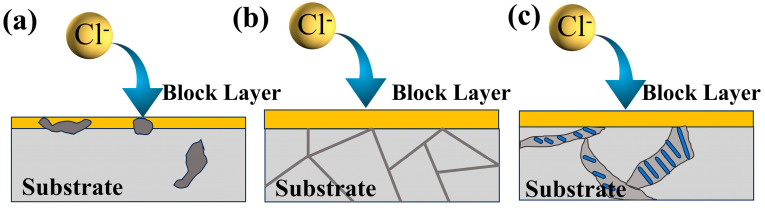
Corrosion mechanism (**a**) cast-a-12h (**b**) T4-a-12h, (**c**) T6-a-12h.

Based on the results of the electrochemical and hydrogen evolution experiments, T4-a-12h and T6-a-12h exhibit superior corrosion resistance, whereas Cast-a-12h demonstrates relatively poorer corrosion resistance. As shown in the microstructure of the coatings in Figure 5a and Figure 6a, the coating on the α-Mg surface is thicker, while the LDH coating on the β-Mg_17_Al_12_ surface is thinner, indicating that the coarse, discontinuous β-Mg_17_Al_12_ phase, which is inlaid within the coating, disrupts the integrity of the coating. The interface between the β-phase and the coating facilitates Cl^−^ penetration in the NaCl solution, leading to the degradation of the coating, as shown in Figure 14a. In the polarization curve results shown in Figure 8c, Cast-a-12h exhibits lower LDH polarization resistance and a higher corrosion current. In the electrochemical impedance spectroscopy (EIS) results, as shown in Figure 9, the smaller capacitive arc and lower modulus impedance both indicate the poor corrosion resistance of this incomplete LDH. However, the polarization curve results in Figure 8 also demonstrate that T4-a-12h and T6-a-12h exhibit higher corrosion potentials and lower corrosion currents. In the EIS results, T4-a-12h and T6-a-12h show larger capacitive arcs and lower coating layer impedance, indicating that both T4-a-12h and T6-a-12h have excellent corrosion resistance due to their complete and uniformly thick coatings, as shown in Figure 5(b,c) and Figure 6(b,c). This provides stronger protection for the magnesium alloy by effectively preventing chloride ions (Cl^−^) from penetrating the substrate, as shown in Figure 14(b,c). This is consistent with the results of the hydrogen evolution reaction (HER) experiment, further confirming the important role of LDH coatings in enhancing the corrosion resistance of magnesium alloys. These experimental results collectively highlight the importance of uniform and dense LDH coatings in corrosion protection of magnesium alloys.

## 5. Conclusions

This study successfully prepared LDH coatings on the surface of AZ91 magnesium alloy substrates in three different heat-treated conditions by employing single cation (containing only Al^3+^) and double cation (Mg^2+^ and Al^3+^) co-precipitation combined with a hydrothermal method. The experimental results and analysis indicate that the microstructure of the magnesium alloy significantly influences the growth process, microstructure, and corrosion resistance of the LDH coatings.

1. In Cast-AZ91, the coarse β-phase exhibits strong galvanic corrosion with Mg, accelerating the dissolution of Mg and promoting the initial formation of the LDH coating. As the reaction time increases, the coating grows both inward and outward, and the large β-phase embedded in the coating disrupts its integrity. In T6-AZ91, fine, dispersed β-phases are formed, while in T4-AZ91, a single solid solution eliminates the negative impact of the coarse β-phase, facilitating the formation of a uniform and compact LDH surface coating during the self-corrosion process.
2. The LDH coatings on T6-AZ91 and T4-AZ91 exhibit superior corrosion resistance compared to those on Cast-AZ91. This is attributed to the coarse β-phase in Cast-a-12h, which compromises the integrity of the coating, making the weaker areas more susceptible to Cl^−^ attack. In contrast, the coatings on T6-AZ91 and T4-AZ91 are more uniform and compact, significantly enhancing the corrosion resistance of the magnesium alloy.
3. Hydrolysis diagrams of Al and Mg were constructed under different pH conditions, allowing for the prediction of LDH chemical reactions under varying microstructural conditions, thereby improving understanding of the reaction mechanisms of LDH in different pH environments.

## Figures and Tables

**Table 2:** Fitting results of the potentiodynamic polarization curves of substrate and Mg-Al LDHs coatings.

Samples	E_corr_ [V/SCE]	I_corr_ [A·cm^−2^]	b_a_ [mV·dec^−1^]	b_c_ [-mdec^−1^]	R_p_ [Ω·cm^−2^]
Cast-AZ91	−1.44	2.18 × 10^−6^	19.83	90.14	2.01 × 10^3^
T4-AZ91	−1.49	2.82 × 10^−5^	38.00	174.13	3.77 × 10^2^
T6-AZ91	−1.46	4.30 × 10^−6^	45.00	120.00	1.04 × 10^3^
Cast-a-20min	−1.25	9.5 × 10^−8^	64.79	99.25	1.80 × 10^5^
T4-a-20min	−1.38	2.39 × 10^−6^	35.33	137.46	5.07 × 10^3^
T6-a-20min	−1.28	5.83 × 10^−8^	32.162	79.46	1.70 × 10^5^
Cast-a-12h	−1.33	1.52 × 10^−8^	262.3	148.22	2.72 × 10^6^
T4-a-12h	−1.2	2.61 × 10^−9^	116.99	98.129	8.86 × 10^6^
T6-a-12h	−1.19	3.41 × 10^−9^	130.89	120.44	7.95 × 10^6^

**Table 3:** Fitting results of the EIS data for different synthesis times of LDH coatings.

Samples	*CPE_f_*		*R_f_* [Ω·cm^2^]	*C_f_* [F·cm^−2^]	*CPE_dl_*		*R_ct_* [Ω·cm^2^]	*C_dl_* [F·cm^−2^]	*R_L_* [Ω·cm^2^]	*L* [H·cm^−2^]	χ^2^
	*Y*_0_ [Ω^−1^cm^−2^s^n^]	*n*			*Y*_0_ [Ω^−1^cm^−2^s^n^]	*n*					
Cast-AZ91	9.41 × 10^−6^	0.93	6784	7.31 × 10^−6^	6.90 × 10^−4^	0.92	3608	0.00087	3823	6605	5.96 × 10^−3^
T4-AZ91	1.24 × 10^−5^	0.92	1551	7.98 × 10^−6^	6.06 × 10^−5^	0.90	1547	3.71 × 10^−5^	20	129.6	9.60 × 10^−3^
T6-AZ91	6.84 × 10^−6^	0.93	6376	6.08 × 10^−6^	2.45 × 10^−5^	0.96	2230	2.75 × 10^−5^	7872	4.923	6.34 × 10^−3^
Cast-a-20min	3.8 × 10^−6^	0.93	3.33 × 10^4^	3.12 × 10^−6^	3.65 × 10^−4^	0.61	1.4 × 10^4^	1.09 × 10^−3^	729.4	31,371	5.95 × 10^−3^
T4-a-20min	3.53 × 10^−6^	0.97	6172	3.20 × 10^−6^	2.6 × 10^−4^	0.40	1680	7.47 × 10^−5^	10	995.7	5.82 × 10^−2^
T6-a-20min	3.63 × 10^−6^	0.94	1.94 × 10^4^	3.14 × 10^−6^	1.59 × 10^−4^	0.50	1.2 × 10^4^	2.49 × 10^−4^	4119	1330	9.11 × 10^−3^
Cast-a-12h	3.23 × 10^−7^	0.76	7.06 × 10^4^	9.8 × 10^−8^	1.11 × 10^−5^	0.77	8.48 × 10^7^	3.47 × 10^−4^	---	---	3.03 × 10^−3^
T4-a-12h	8.05 × 10^−8^	0.74	1.0 × 10^5^	1.59 × 10^−8^	9.64 × 10^−6^	0.77	8.11 × 10^8^	6.7 × 10^−5^	---	---	6.4 × 10^−3^
T6-a-12h	1.1 × 10^−7^	0.73	9.59 × 10^4^	2.1 × 10^−8^	1.01 × 10^−5^	0.79	8.54 × 10^8^	9.68 × 10^−5^	---	---	5.6 × 10^−3^

## Data Availability

The original contributions presented in this study are included in the article. Further inquiries can be directed to the corresponding author.
